# Considerations from the risk of bias perspective for updating Cochrane reviews

**DOI:** 10.1186/s13643-015-0122-3

**Published:** 2015-10-06

**Authors:** Alain D. Mayhew, Monisha Kabir, Mohammed T. Ansari

**Affiliations:** Knowledge Synthesis Group, Clinical Epidemiology Program, Centre for Practice-Changing Research (CPCR), Ottawa Hospital Research Institute, Ottawa, Canada; Biology Programme, University of Ottawa, Ottawa, Canada; School of Epidemiology, Public Health and Preventive Medicine, Faculty of Medicine, University of Ottawa, Ottawa, Canada

**Keywords:** Updating, ‘Risk of bias’, Cochrane methods, Systematic reviews

## Abstract

Authors of Cochrane reviews are expected to update their reviews every 2 years. The updating process helps to ensure that reviews are current and include recent evidence. However, the updating process is time-consuming for authors, particularly when Cochrane methods evolve and authors are required to revisit some of the originally included studies.

The Cochrane Collaboration's ‘Risk of bias’ tool is a mandatory component of Cochrane reviews, providing an assessment of the potential biases of included studies. The tool has been modified most recently in 2011, and the expectation is that new versions will continue to be produced and utilised in all Cochrane reviews. In this commentary we discuss, in the context of updating scenarios that are likely to be encountered, the potential options systematic review authors may have recourse to when the Cochrane Collaboration's ‘Risk of bias’ tool has been modified between the original review and its update. We recommend that authors who are updating reviews should revise their original assessments of included studies using the most recent version of the risk of bias tool. Despite the increased workload, use of the most recent version of the tool facilitates consistency of methods and reporting both across and within reviews, and ensures currency to the methodological rigour.

## Background

The Cochrane Collaboration publishes systematic reviews in the Cochrane Database of Systematic Reviews (CDSR). Cochrane reviews, like non-Cochrane ones, are expected to be current; and when authors agree to take on and complete a Cochrane review, the expectation is that the review will be updated every 2 years. This update must include a new search for studies, and if new studies are identified, they must be screened and the relevant data extracted. A new synthesis including both previously identified studies and newly found studies is then published [1].

Updating systematic reviews is a challenge both for review authors and others involved with the publication process, such as peer reviewers and editors. Cochrane updates are given a new citation as long as they include a new recent search and a screening process for articles [2]. There is a substantial amount of time and resources required to complete any systematic review update. The novelty of the original publication no longer exists, and it is possible that the members of the authorship team no longer collaborate or some team members may have moved on to different areas of research. In some cases, a new research team takes over the review and carries out the update. Most of the research on updating reviews has focused on signals identifying the need to update [3–8] and reasons and methods for updating [9, 10]. Although authors agree to update reviews when they take on the project initially, approximately only 20 % of Cochrane reviews are updated every 2 years [11].

One of the challenges of updating a review is incorporating new methods. The field of scientific writing continues to evolve [12, 13]. The Cochrane Collaboration has 16 method groups, which specifically target and develop the methods used in the conduct and reporting of systematic reviews. In this commentary we discuss, in the context of updating scenarios that are likely to be encountered, the potential options systematic review authors may have recourse to when the Cochrane Collaboration’s ‘Risk of bias’ (RoB) tool has been modified between the original review and its update. We recommend that in order to keep a review current, authors need to not only incorporate new evidence but also utilise the most up-to-date methodological guidance.

### Recent history of Cochrane risk of bias assessment for randomised trials

There have been significant changes to the Cochrane RoB tool in the past 10 years. The three most recent versions are described below and summarised in Table 1.

**Table 1:** A comparison of Cochrane risk of bias approaches since 2006

Risk of bias version	Biases assessed	Specific domains	Mandatory component in Review Manager software
2006	Recommendation was made to assess selection bias (allocation concealment), performance bias, attrition bias and detection bias for every study.		Selection bias (specifically allocation concealment) was incorporated into Review Manager and scored as ‘adequate (A), unclear (B), inadequate (C) or that allocation concealment was not used (D)’. Other biases were not mandatory and likely not consistently assessed. No justification of judgement was required.
2008	Development of a tool for assessing risk of bias. Four biases as above, with ‘other bias’ category for authors to consider additional biases. No limit on the number of additional biases authors can identify.	Sequence generation, allocation concealment, blinding, incomplete outcome data, selective outcome reporting and other domains which authors could add.	Assessment of six domains within five biases mandatory. Judgement of ‘Yes’ (low risk), ‘No’ (high risk) or ‘Unclear’. Justification of judgement mandatory for each domain for each study.
2011	2008 tool modified but no new biases added.	As per 2008 version, except blinding divided into two domains, one related to blinding of participants and personnel, and the other related to outcome assessors.	Judgements changed to ‘High risk’, ‘Low risk’ or 'Unclear risk’. Clarification of which category of bias domain refers to and additional guidance for other risk of bias.

According to the Cochrane Handbook for Systematic Reviews of Interventions (referred to as the Handbook below) published in 2006, the recommendation was made to assess selection bias, performance bias, attrition bias and detection bias for every study [14], but only allocation concealment was a mandatory component of the RoB table in the Review Manager software used at that time. There was no requirement for a description of justification of any decisions and there was likely inconsistency in both the decision-making and the content of this reporting.

In the 2008 version of the Handbook, there was a major revision to the RoB tool [15]. This version of the tool included five bias categories and six mandatory components. There were options for considerations of outcome-specific components. The assessment of each component was done using ‘Yes’ , ‘No’ or ‘Unclear’ categories. For each judgement, the review author was required to include a justification based on text within the review and where possible, a direct quote.

In 2011, the Handbook was updated and further changes were made to the RoB tool [16]. To distinguish between performance bias affecting actual outcomes and detection bias in their inaccurate estimation, this version of the RoB tool included a recommendation to separate assessment of blinding into two components, one for study participants and intervention providers, and one for outcome assessors, respectively. Judgements were changed to ‘High risk’ , ‘Low risk’ or ‘Unclear risk’ , but the requirement to justify all judgements remained.

In 2008, the Cochrane Handbook suggested that all Cochrane reviews include a summary of findings (SoF) table [17]. The risk of bias assessment influences the reporting of the quality of evidence of specific outcomes in the SoF table. Previous research has demonstrated that only 9 % of updated Cochrane reviews have a change in conclusions compared to the original review [18]. Changes in the new risk of bias assessments leading to the potential changes of the content of the SoF tables could influence the overall conclusions. Further, robustness of meta-analytic estimates and exploration between study heterogeneity in estimates of effects is often explored in sensitivity analyses—repeat primary meta-analyses limited to subgroups of studies. Changes in study level risk of bias assessments have the potential to change estimates of effects in sensitivity analyses and explain heterogeneity that was previously unexplained.

Given the slow rate of updating, it is common for newly published review updates to require a full reassessment of studies included in earlier versions. For example, if the original version of a review was published in 2004 and updated in 2014, then all originally included studies would require reassessment using the 2011 RoB tool for methodological consistency. In 2014, over 400 reviews were updated in CDSR. Over 75 % of these updates were published as original reviews prior to January 2011, indicating that at some point, authors should have reassessed the previously included studies using a second, more current RoB tool [19].

The 2011 version of RoB improves the transparency of published reviews and the framework for assessing risk of bias [20]. Both the additional mandatory categories and judgements for decisions provide an uncomplicated method for authors to judge the impact of the risk of bias on the review results. Depending on when the previous version of a review was published, it is clearly a substantial amount of work for authors of updates to revisit all of the included studies in the previous publication, reassess their risk of bias and adjust the results accordingly. Though the application of the Cochrane RoB tool is required as per the Methodological Expectations of Cochrane Intervention Reviews [21], only 74 % of Cochrane review groups reported enforcing the application of the tool to all randomised controlled trials when updating a systematic review [22].

## Discussion

### Possible approaches

There are four different risk of bias scenarios to consider that reviewers may encounter when updating reviews. For our purposes, the decision-making has been broken down into whether studies were included in the original review, and subsequently, whether any additional eligible studies were found in the updated search. The different scenarios are presented with a rationale for, and implications of, each decision. The scenarios below apply to reviews for which the research questions and 'population-intervention-comparison-outcome', or PICO, framework remain essentially unchanged. When important changes to the research questions or framework are required (e.g. new important harms have been identified, new population and disease indications have emerged, comparator(s) has changed because another treatment(s) has now also diffused into current practice, etc.), a new protocol must be developed to undertake an entirely new review rather than update an existing one. Decisions at this time must be made whether to proceed with an update or to publish a new review with the new parameters.

### 1. Updating reviews where previous version(s) had no included studies

- *No new eligible studies identified in the updated search*

After complete searching and screening according to predetermined criteria, there are still some systematic reviews that have no included studies, commonly referred to as empty reviews [23, 24]. If no new studies are found in the search for the updated review, and the review remains empty, then there is obviously no need to apply a RoB tool.

b. *One or more eligible studies found in the updated search*

If one or more new studies are judged to be eligible for inclusion in the review from a new recent search, the clear choice would be to use the current 2011 RoB tool. The authors would be required to do full data extraction of the newly included studies and as such, would also be expected to perform a full assessment of the risk of bias.

### 2. Updating reviews where previous version(s) had included studies

- *No new eligible studies identified in the updated search*

**Fig. 1:**
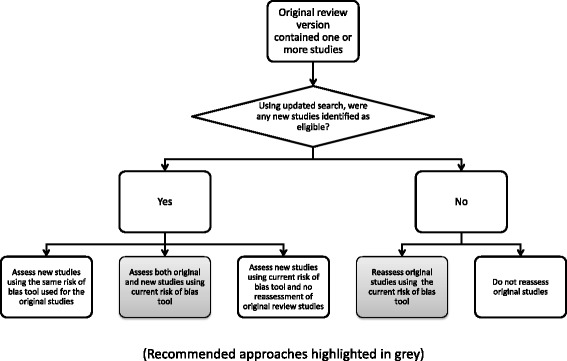
Options for assessing risk of bias when updating reviews with one or more original studies

One could argue in this case that redoing RoB assessments of previously included studies is not necessary (Fig. 1). The 2011 Cochrane Handbook does not strongly recommend the reassessment of included studies when no new eligible studies are found [25]. However, the only solid rationalization for not re-evaluating previously included studies is a minimization of the work required. The fact that the review will be labelled, published and cited as updated implies that the methods used are current. The best approach is for the authors to reassess all the original studies using the current RoB tool. As described above, authors will receive a new citation for the update, even if there are no new studies included. The rationale is that updating of reviews is a holistic undertaking in which all aspects need to be current and up-to-date; not only searches but also methodological approaches to critical appraisal and synthesis of evidence. An argument may be made that empiric evidence does not exist demonstrating meaningful differences between the current and earlier versions of RoB tool. Nonetheless, revisions leading to the current tool have been made based on author and user input. The endorsement of the current tool by the Cochrane Collaboration is reason enough why all studies within a review should be assessed using the most recent tool.

However, there are important challenges in incorporating the most current risk of bias tool into an update of a review. Depending on the age of the review, it is possible that the authors have performed a risk of bias assessment on all studies in the previous version of the review using an older version of the tool. One could argue that older studies may be subject to a time lag bias as authors of older studies may have used acceptable methods at the time the study was conducted, but unfortunately, those studies would be graded as higher risk of bias based on current standards. One example of this is trial registration or protocol publication (or availability) to detect or clarify the absence of selective outcome reporting. To comply with the U.S. Food and Drug Administration Modernization Act (FDAMA) of 1997, clinicaltrials.gov was launched in 2000 as a trial registration platform [26]. Trial registration became an ICMJE requirement in 2005 [26] and was later mandated under the U.S. Food and Drug Administration Amendments Act (FDAAA) of 2007 [27]. Prior to 2005, there was no easy process for trial registration, and it was very rare for protocols to be published for individual trials. Therefore, older studies are much more likely to receive an ‘unclear’ RoB assessment of selective outcome reporting compared with trials published after 2007. Granted this may also be true for older included studies included in the first version of a review, but for updating, we are recommending authors reassess the reviews which involves additional work and may impact on the results. Furthermore, authors of older studies would report their findings according to the journal requirements which may have been different 10 or more years ago when the Consolidated Standards of Reporting Trials (CONSORT) statement was less widely adopted [28].

When writing Cochrane reviews, authors report that a RoB criterion is ‘unclear’ when the information is not available. One approach to dealing with the lack of clear reporting of information is to contact individual study authors. In principle, this approach seems logical and very useful. Unfortunately, there are two major limitations. First of all, it can be very difficult to track down author teams for individual studies. Researchers change institutions, research interests change and teams split up; as a result, study processes which may or may not contribute to RoB may be difficult to recall or report accurately. Secondly, there is evidence that contacting study authors may lead to overly positive answers. In a survey of 104 trialists, using direct questions about blinding with named categories of trial personnel, 43 % responded that the data analysts in their double-blind trials were blinded, and 19 % responded that the manuscript writers were blinded [29]. This is unlikely to be true, given that such procedures were reported in only 3 and 0 % of the corresponding published articles, and that they are very rarely described in other trial reports. Updating a review is unlikely to change judgements on a specific criterion which was judged to be ‘unclear’ , as it will be even more difficult to obtain information from authors because at least two more years will have passed since the included study was published.

b. *One or more studies found in the updated search*

The consistency of methods and reporting within a review is an important issue. Review authors will likely choose to assess all the studies similarly, either using a previous RoB tool or the more recent version. However, as argued above, the best practice approach is for review authors to apply the 2011 RoB tool to all included studies, both those included in the previous review publication and the newly identified studies for inclusion to maintain review currency and associated face validity, regardless of the number in each category. The discussion above is relevant for an update with new studies, and the added concern of consistency within a review makes it very difficult to rationalise using an old tool for newly found studies. It is worth noting that if the search strategy is modified, there may be studies identified which should be included that were published prior to the original publication but not captured in the previous version of the review. The lack of reporting guidelines and difficulty contacting authors would be an issue for these newly identified but older studies, but they would still be included in the review and require a full assessment including risk of bias.

This commentary discusses an important methodological consideration associated with the updating of systematic reviews that is currently not being enforced universally as authors update their systematic reviews. We recommend that systematic review authors should not only update their review with new evidence but also ensure that the methods used are current. This is particularly relevant for the study risk of bias assessment using the Cochrane RoB tool which has gone several iterative revisions over the past 10 years.

Although we believe the recommendations are sound, there are some implications. The original version of a review would have described in the methods the techniques that would have been used for the RoB assessment. Theoretically, authors who use the newer RoB tools for an update would not be following their protocol. It would be very important for authors to document how the RoB assessment has changed. Cochrane authors are required to describe differences between protocol and review, and the use of a more recent RoB tool in an update should be clearly documented.

For a new review, in the process of writing the protocol, the review authors are expected to consider and clearly document how they will incorporate the risk of bias assessments into the results. These same decisions and documentation should be followed when performing an update. Authors should refer to the most recent version of the Cochrane Handbook for guidance and outline in the methods and any changes to the approach of incorporation of risk of bias assessment into the results. Authors should also be prepared for the possibility that results (including judgement about the reviewers’ confidence in the estimates of effects or quality of evidence) and conclusions may change by incorporating a newer RoB tool into the methods. Besides the actual shift in the magnitude and direction of the estimates of effects with new evidence, using a more current risk of bias tool could also potentially change the assessment of the quality of evidence, overall conclusions or both.

It is clear that using the most recent RoB tool for studies that were assessed in an earlier version of a review, substantially increases the workload. However, the improvements in the methods and the consistency across all Cochrane reviews are critical. As discussed above, authors receive full citation credit for an update, and therefore should meet any expectations of the publishing body.

Implementation of these recommendations will continue to be a challenge. There are new MECIR standards being developed for updating Cochrane reviews. Inclusion of our recommendations in the standards and in the Handbook will likely increase the chance of the recommendations being followed.

There will continue to be new developments and major revisions to the RoB tool used in Cochrane reviews. There is a new RoB tool recently launched for non-randomised studies [30]. New biases and new assessment tools are being developed for randomised controlled trials as well, and there will continue to be a need for authors of updates to consider the best approach. It is likely that other review components such as summary of findings tables, searching and statistical analysis will all continue to evolve and lead to additional work for authors to consider when updating reviews. These considerations can also potentially apply to other methodological aspects of evidence syntheses as corresponding guidance evolves—for example, approaches to meta-analysing sparse data, subgroup analyses and recommendations for assessment of publication bias.

## Conclusion

Updating Cochrane reviews, and non-Cochrane reviews, remains a challenge for many reviewers. Nonetheless, using the newest version of the RoB tool for all included studies, even those studies included in the previous versions of the review, is the recommended approach. Authors and readers should be well aware of the challenges newer methods of risk of bias assessments present when updating Cochrane reviews.

## Abbreviations

CDSR: Cochrane Database of Systematic Reviews
CONSORT: Consolidated Standards of Reporting Trials
RoB: ‘Risk of bias’
SoF: 'Summary of findings'
